# Knowledge and attitudes toward the use of anabolic–androgenic steroids among physical education university students: a cross-sectional study from Palestine

**DOI:** 10.1038/s41598-024-52787-w

**Published:** 2024-01-25

**Authors:** Suhaib Hattab, Bashar Saleh, Laith Qasarweh, Mahmoud Draidi, Sa’ed H. Zyoud

**Affiliations:** 1 Physiology, Pharmacology and Toxicology Division, Department of Biomedical Sciences, Faculty of Medicine and Health Sciences, An-Najah National University, Nablus, Palestine; 2 Department of Physical Education, An-Najah National University, Nablus, Palestine; 3 Department of Medicine, Faculty of Medicine and Health Sciences, An-Najah National University, Nablus, Palestine; 4 Department of Clinical and Community Pharmacy, College of Medicine and Health Sciences, An-Najah National University, Nablus, 44839 Palestine; 5 Poison Control and Drug Information Center (PCDIC), College of Medicine and Health Sciences, An-Najah National University, Nablus, 44839 Palestine; 6 Clinical Research Center, An-Najah National University Hospital, Nablus, 44839 Palestine

**Keywords:** Health care, Health occupations, Medical research

## Abstract

The use of androgenic–anabolic steroids (AASs) has increased in recent years, especially among athletes, due to their effect on body shape and performance. These agents could have serious side effects on this highly susceptible population, which tends to use these substances frequently to promote muscle growth and physical performance. Therefore, this study aimed to evaluate the knowledge and attitudes toward the use of anabolic androgenic steroids among physical education university students in Palestine. A cross-sectional study of physical education students from November 2020 to January 2021 was conducted using an electronic questionnaire. The main outcome was to measure the level of knowledge and use of the AAS. A total of 380 students were included. The mean age of the students was 21 years (SD = 4.2), and the study participants were distributed almost equally according to sex. Approximately a quarter of the students were smokers. Eighty percent (80%) of the study participants were from the West Bank, while the remaining 20% were from Jerusalem and 48 territories. Furthermore, most of the students lived with their families; half lived in cities, approximately 152 (40%) lived in villages, and only 29 (8%) lived in camps. The average level of knowledge of the AAS was 2.95 out of 8 (37/100), with a median of three. Furthermore, only 36 (10%) of the participants had satisfactory knowledge, scoring 80% or more. Regarding the use of AAS, 11 (2.9%) participants, all males, confirmed that they were currently using AAS. Additionally, approximately 28 (7%) had previously used them, while 30 (8%) planned to try them in the future. Overall, 221 (58%) patients were confirmed to use vitamins and minerals. Our study showed that most of the participants had a substantial lack of information on the potential side effects of AAS, while the level of use was comparable with that of other populations.

## Introduction

Androgenic–anabolic steroids (AASs) are synthetic derivatives of the male hormone testosterone, one of the most important endogenous androgens. Androgens are important hormones for the expression of the male phenotype. They play crucial roles during male sexual differentiation, as well as during the development and maintenance of secondary male characteristics and the initiation and maintenance of spermatogenesis^1^. Pharmacologically, anabolic steroids are used to treat cachexia associated with chronic diseases and to address muscle mass in elderly individuals^2^.

AAS can exert strong effects on the human body and may benefit athletic performance. For several decades, athletes and bodybuilders have recognized that anabolic steroids can promote muscle growth and strength and increase athletic performance, an effect that is particularly evident in women^3^. In recent years, the use of the AAS has been increasing, especially among athletes. According to a meta-analysis that included 271 studies, the overall prevalence was 3.3%. The prevalence was greater for men than for women. Furthermore, the incidence of AAS varied by region, with the highest incidence occurring in the Middle East (21.7%), followed by South America, Europe, North America, Oceania, Africa, and Asia (4.8%, 3.8%, 3.0%, 2.6%, 2.4%, and 0.2%, respectively)^4^.

Several studies have investigated the adverse effects of AAS; most of these are dose related. Notably, sudden death and damage to vital organs have been reported^5–7^. Cardiovascular complications included myocardial infarction and atrial fibrillation^8–10^. Furthermore, kidney function can be altered, as a recent study showed that bodybuilders who used AAS for several years developed proteinuria and a severe reduction in kidney function^11^. In addition to organic diseases, the use of the AAS has been associated with psychiatric complications, including violent behavior and suicide^12,13^.

The impact of AAS on the reproductive system is of particular concern. A meta-analysis of 33 studies with 3879 participants revealed significant reductions in luteinizing hormone, follicle-stimulating hormone, and endogenous testosterone levels during AAS intake. After discontinuing AAS, the serum Gn levels gradually returned to baseline values within 13–24 weeks, while the serum T levels remained lower than baseline. Her serum testosterone levels decreased at 16 weeks after the discontinuation of AAS. In addition, the abuse of AASs results in structural and functional changes in sperm, a reduction in testicular volume, gynecomastia, clitoromegaly, menstrual irregularities, and subfertility^14^.

This study aimed to evaluate the knowledge and attitudes toward the use of anabolic androgenic steroids among physical education university students in the northern region of the West Bank, Palestine. This understanding will help direct efforts and make changes to policies intended to address the issue of AAS misuse from the point of view of the healthcare system.

## Methods

### Study setting

The study was carried out in the Department of Physical Education at An-Najah National University in Palestine. This department's program aims to prepare students in both academic and practical matters and to provide the community with qualified graduates with a bachelor’s or master’s degree in physical education. Students receive advanced educational, practical, and research skills; experience in training clubs and fitness centers; and administrative work in various sports institutions through personal refinement to meet this distinctive role that the faculty aspires to provide the community with all of its sports.

### Population and sample size

The department has approximately 492 students who are distributed throughout all governorates, including the West Bank, Jerusalem, and Palestinians living in the occupied area of Palestine. Of those, 418 students were enrolled in a bachelor's programme (75, 114, 112, and 117 in the 1st, 2nd, 3rd, and 4th years, respectively), while 74 were enrolled in a master’s programme. The sample size for this study was initially set at 216 using the Raosoft electronic sample size calculator. This calculation factored in a 5% margin of error, a 95% confidence level, and assumed a 50% response rate from a population of 492 students. However, to enhance the statistical power of the study and minimize errors, the sample size was ultimately increased to 380.

### Study design

This was a cross-sectional cohort study that was conducted from November 2020 to January 2021. The participants were all students enrolled in the Department of Physical Education at both the bachelor’s and master’s levels. Any student willing to participate was included in this study. The study was explained to all participants before they completed the questionnaire. Patients with incomplete responses were excluded. The questions and answers were written in Arabic and then translated into English by the researchers.

### Study tool

The study tool used was an electronic questionnaire generated via Google^®^ documents, which was shared as a link on the Department of Physical Education website. This valid questionnaire was used in previous research by Althobiti et al*.*^15^. All the students in the department were invited to complete the questionnaire only once. The questionnaire contained four sections (Additional File 1). The first section included a consent form that clearly explained the study objectives and emphasized that all the information collected would be kept confidential and used for research purposes only. Should the student agree to participate? The second section collected students' sociodemographic characteristics, such as age, sex, height, weight, smoking status, place of residency, place where the student was born, academic level, marital status, and monthly salary income. Additionally, information on chronic diseases or current medication use was collected. The third section included eight (8) questions to measure the students' knowledge about AAS. These questions inquired whether the use of AAS could cause acne, hair loss, infertility, and cardiovascular disease and whether the cessation of these agents could cause depression, muscle atrophy, or decreased physical activity. The last question was about the legality of the use of these agents for bodybuilding. The fourth section contained questions regarding the use of the AAS. These questions inquired about the previous, current or future use of AASs and the source of information about these agents. In addition, the questionnaire asked them whether users would advise other colleges to use AASs. An additional question regarding the use of vitamins and minerals before asking about AAS was asked to ensure that participants knew what was meant and that it differed from minerals and vitamins. Satisfactory knowledge of the AAS was identified as having a score of 80% or more in the third section that measured knowledge about the AAS.

### Statistical analyses

Statistical analyses were performed using the statistical package SPSS Version 22 (IBM Corporation, Armonk, NY, USA). Descriptive statistical analysis was used to determine the frequency, mean, median, and standard deviation. In addition, nonparametric tests such as the Kruskal‒Wallis test and the Mann‒Whitney test were used to compare the level of knowledge among different groups. Considering a confidence interval of 95%, a p value less than 0.05 was considered to indicate statistical significance.

### Ethics approval and consent to participate

The study was carried out according to the regulations and ethics of An-Najah National University (Nablus-Palestine) and in accordance with the Declaration of Helsinki. The study received ethical approval from the Institutional Review Board (IRB) of An-Najah National University (Protocol # 03-2020). Each participant provided her electronic informed consent prior to participating in the study. We confirm that all the experiments and methods were performed in accordance with the relevant guidelines and regulations.

## Results

### Sociodemographic characteristics of the study participants

The response rate for the study questionnaire was 77% (380 of 492). Study participants were distributed almost equally according to sex, with a mean age of 21 years (SD = 4.2). Two-thirds of the students had a normal body mass index; 82 (21%) were overweight, and only 20 (5%) were obese. Approximately one-fourth of the students were smokers. Approximately 300 (80%) of the study participants were from the West Bank, and the remaining 80 (20%) were from Jerusalem and 48 territories. Furthermore, most of the students lived with their families. Approximately 40% of the participants lived in cities, only 8% lived in villages, and only 8% lived in camps (Table 1).

**Table 1:** Characteristics of the study participants (n = 380).

	Number (%) or mean ± SD
Age, mean	20.7 ± 4.2
Gender	
Male	176 (46.3)
Female	204 (53.7)
BMI	
Underweight, BMI is less than 18.5	16 (4.2)
Normal weight, BMI is 18.5 to 24.9	261 (68.7)
Overweight, BMI is 25 to 29.9	82 (21.6)
Obese, BMI is 30 or more	20 (5.3)
Smoking status	
Smoker	100 (26.3)
Nonsmoker	280 (73.7)
Current residency of student family	
Village	152 (40)
City	199 (52.4)
Camp	29 (7.6)
Place of residence (born and raised)	
West Bank	300 (78.9)
Jerusalem	36 (9.5)
Palestinians living in the occupied area of Palestine	44 (11.6)
Place of current residence during university study	
Alone in students apartment	11 (2.9)
With other students at students apartment	13 (3.4)
With the family	356 (93.7)
Academic level	
Bachelor	341 (89.7)
Master	39 (10.3)
Academic level for bachelor students	
First year	104 (27.4)
Second year	89 (23.4)
The Third year	95 (25)
Fourth year	92 (24.2)
Marital status	
Married	22 (5.8)
Single or divorced	358 (94.2)
Monthly salary income	
Low: less than 2000 NIS	48 (12.6)
Moderate: 2001–5000 NIS	174 (45.8)
High: 5001–10,000 NIS	96 (25.3)
Very high: More than 10,000 NIS	51 (13.4)
Chronic diseases	5 (1.3)
Current chronic medication	32 (8.4)

*NIS* New Israeli shekel (1 NIS = 0.31; U.S. dollars), *BMI* body mass index.

In general, most of the students were single. Half of the students had a moderate monthly salary income, while a quadrant had a high income, and the latter quadrant was distributed between low and very high income. Generally, the students were apparently healthy; only 1% had chronic diseases, and few had taken chronic medications (Table 1).

### Level of knowledge about AAS among participants and relation to sociodemographic factors

The average level of knowledge about the AAS was 2.95 out of 8 (37/100), with a median of three. Furthermore, only 36 (10%) participants had satisfactory knowledge, scoring 80% or more. The questions with the highest level of knowledge were those asking whether AAS increases the risk of CVD and whether cessation of these drugs decreases muscle mass. On the other hand, the questions related to the lowest level of knowledge were about the legality of using these drugs for muscle-building purposes and whether cessation of these drugs would increase the risk of depression. Another important question on the side effects of AASs showed that only approximately 40% of the respondents knew about this side effect.

Regarding the sociodemographic characteristics of the study participants, several factors affected their level of knowledge about AAS. Male sex was associated with a greater knowledge score than female sex was (p = 0.002). Additionally, the current residency of the student family and the place of residence had significantly different levels of knowledge; those residing in camps had a greater level of knowledge than did those living in villages or cities (p = 0.004). Furthermore, students living in the occupied area of Palestine or Jerusalem had a greater level of knowledge than did those living in the West Bank (p = 0.002).

Regarding monthly salary income, participants' knowledge level increased proportionally with increasing income, but no significant differences in knowledge were found among participants with different salary incomes. Furthermore, other factors, such as BMI, smoking status, current residence during university study, academic level, and marital status, were not associated with knowledge level (Table 2).

**Table 2:** Level of knowledge about AAS among participants and relationship to sociodemographic factors.

	Median [Q1, Q3]	Uni-variable analysis	Multivariable analysis
		p value	
Gender			
Male	4.0 [0.0, 6.0]	0.008	0.002
Female	3.0 [0.0, 5.0]		
BMI			
Underweight, BMI is less than 18.5	2.0 [0.0, 4.7]	0.51	
Normal weight, BMI is 18.5 to 24.9,	[0.0, 5.0]		
Overweight, BMI is 25 to 29.9	3.0 [0.0, 5.0]		
Obese, BMI is 30 or more	0.0 [0.0, 3.7]		
Smoking status			
Smoker	4.0 [0.0, 5.0]	0.54	
Nonsmoker	3.0 [0.0, 5.0]		
Current residency of student family			
Village	2.5 [0.0, 5.0]	0.01	0.004
City	3.0 [0.0, 5.0]		
Camp	5.0 [4.0, 7.0]		
Place of residence (born and raised)			
West Bank	2.0 [0.0, 5.0]	0.00	0.002
Jerusalem	4.5 [3.2, 6.0]		
Palestinians living in the occupied area of Palestine	4.0 [1.2, 5.7]		
Place of current residence during university study			
Alone in students apartment	0.0 [0.0, 5.0]	0.397	
With other students at students apartment	4.0 [2.5, 5.0]		
With the family	3.0 [0.0, 5.0]		
Academic level			
Bachelor	3.0 [0.0, 5.0]	0.64	
Master	4.0 [1.0, 6.0]		
Academic level for bachelor students			
First year	2.0 [0.0, 5.0]	0.276	
Second year	[0.0, 5.0]		
The Third year	4.0 [0.0, 5.0]		
Fourth year	2.0 [0.0, 5.0]		
Marital status			
Married	4.0 [0.7, 5.2]	0.189	
Single or divorced	3.0 [0.0, 5.0]		
Monthly salary income			
Low: less than 2000 NIS	2.0 [0.0, 4.0]	0.022	0.114
Moderate: 2001–5000 NIS	3.0 [0.0, 5.0]		
High: 5001–10,000 NIS	3.0 [0.0, 5.0]		
Very high: more than 10,000 NIS	4.0 [3.0, 5.0]		

*NIS* New Israeli shekel (1 NIS = 0.31; U.S. dollars), *BMI* body mass index.

### Extent of AAS use among participants

Regarding the use of AAS, 11 (2.9%) participants, all men, confirmed that they were currently using AAS, such as Anavar^®^, Sustanon^®^ and Masteron^®^. This constitutes the prevalence of the use of the AAS among physical education university students in Palestine. Additionally, approximately 28 (7%) had previously used them in the last 12 months, while 30 (8%) were planning to try them in the future. Overall, 221 (58%) patients were confirmed to use vitamins and minerals (Table 3).

**Table 3:** Extent of AAS use among participants.

	N (%)
Previous AAS use	28 (7.4)
West bank	18
Jerusalem	6
48 territories	4
Current AAS use	11 (2.9)
West bank	7
Jerusalem	3
48 territories	1
Future AAS use	30 (7.9)
West bank	26
Jerusalem	2
48 territories	2
Growth hormone use	13 (3.4)
Vitamins and minerals use	221 (58.2)
The source of information about these agents	
Others	135 (34.5)
Internet	117 (30)
Social media	47 (12.1)
Teacher	40 (10.3)
Friends	20 (5.1)
Study friends	9 (2.3)
Doctor or pharmacist	2 (1.3)
TV	1 (0.3)
Whether users would advise other colleges to use AAS	32 (8)

*AAS* anabolic androgenic steroids.

## Discussion

This study examined the knowledge, attitudes, and behaviors of physical education students about the use of AAS. Furthermore, we characterized AAS users among physical education students in Palestine. Despite being aware of the positive effects of AAS on muscle mass and strength, most of the study participants demonstrated a significant lack of knowledge regarding the possible negative side effects of AAS on the different systems of the body.

The findings of this study revealed poor knowledge about AAS among physical education students; only 10% of the respondents had satisfactory knowledge, with 80% or more of the respondents scoring. Furthermore, approximately 10% of the students currently used or used AASs, while approximately 8% of those who planned to use AASs in the future.

Considering that our population is composed of physical education students with no significant differences in knowledge according to their academic degree, additional interventions and teaching about the use and safety of AASs are needed, especially among this population, which could be coaches or teachers in the future.

The results of the study showed that there is a low level of knowledge about side effects. Similar results were reported in Iran, eastern Saudi Arabia, Kuwait, and Brazil^12,16–19^. A study conducted among male fitness centers in Kuwait showed that only 18% of the participants had adequate knowledge of these drugs, possibly because adequate instruction is lacking on the harmful effects of these drugs and because of different advertisements that show the benefits of these drugs on body shape and mass without focusing on serious side effects. The study also showed that students living in camps have better knowledge of the side effects of these drugs; this could be attributed to the fact that these drugs are more prevalent in these areas without supervision^20^.

Our study showed that the Internet and social networks play important roles in increasing the tendency to use AAS. This finding is similar to those of previous studies among Jordanian collegiate students and athletes^21^ and in western Saudi Arabia^22^.

Approximately 3% of our study participants currently use AASs—all men—which is low compared to the findings of other studies where the prevalence was 18%, 23%, and 9% in Eastern SA, Kuwait, and Sweden, respectively^23^. Furthermore, the Riyadh studies conducted by Al Jabri et al. and Al Harbi et al. had high prevalence rates of 30.5% and 29.3%, respectively^24,25^. This could be attributed to the fact that the population in these studies was composed of male gymnasts who were older than our study participants. Compared to Iran, in Jordan, the prevalence use of AASs was 3% and 4% among medical students and college students, respectively, which is similar to the prevalence in our study, where the population of these studies included university students with a mean age of 22 years for the Iranian study and 20 years for the Jordanian study. This may be due to the embarrassment of the participants, who reported that they were using AAS, or to financial problems because college students cannot afford to buy or have access to these drugs.

The main reason for using AAS was to establish better-looking bodies or bodybuilding; similarly, the main motive of most users in studies in Kuwait, Saudi Arabia, and Brazil was bodybuilding.

Approximately more than half of the participants used vitamins and minerals in addition to AAS and this constituent in the studies conducted in the UAE and Greece^26,27^. Regarding monthly salary income, the level of knowledge among participants increased proportionally with increasing income, which is not consistent with the findings of studies conducted by Albaker et al. and Al-Harbi et al. in eastern and Riyadh, Saudi Arabia, respectively, which revealed a negative association between knowledge about and use of AASs and monthly income^25,28^.

### Limitations

Our findings should be evaluated in light of several limitations. First, the use of the AAS among physical education students is likely underestimated because self-reported health practices are vulnerable to social desirability bias. Second, during the ongoing COVID-19 epidemic, social distancing regulations prevented more accurate actual practices. Third, however, the online survey received an adequate response rate. Finally, because the study was performed entirely online and only students with access to social media networks were included, generalizing the findings to the entire student body would be risky.

## Conclusions

This is the first study conducted among Palestinian physical education students on the use of AASs. Despite being aware of the favorable benefits of AAS on muscle growth and strength, the majority of the survey participants indicated a substantial lack of information on the potential detrimental side effects of AAS on various body systems. This information might be useful in directing efforts and modifying policies to address the issue of AAS misuse in the healthcare system.

### Supplementary Information

Supplementary Information.

## Data Availability

Due to privacy, the data sets used and/or analyzed during the current study are available from the corresponding author upon reasonable request.

## Abbreviations

AAS: Androgenic–anabolic steroids
SPSS: Statistical Package for the Social Sciences
IRB: Institutional Review Board
BMI: Body mass index
SA: Saudi Arabia
UAE: United Arab Emirates
SD: Standard deviation
