# Supplementary material for: Knowledge and attitudes toward the use of anabolic–androgenic steroids among physical education university students: a cross-sectional study from Palestine
Source: Sci Rep. 2024 Jan 25;14:2146. doi: 10.1038/s41598-024-52787-w (PMC10810783; doi:10.1038/s41598-024-52787-w)
Supplement: Supplementary file 1 — Supplementary Information. [file 41598_2024_52787_MOESM1_ESM.docx]

**Additional File 1: Study questionnaires.** This is the final version of the English version that was used to assess knowledge and attitude towards the use of anabolic-androgenic steroids among physical education university students in Palestine.

**English version**

Dear participant,

The purpose of this questionnaire is to identify and shed light on the level of awareness and consumption of anabolic-androgenic steroids among physical education university students. Additionally, we aim to identify the factors influencing this consumption, strategies to address them, and the extent of their impact on students' lives. We kindly request that you answer the following questions.

Please note that the information you provide will remain confidential and will be used solely for scientific research purposes. We appreciate your cooperation in advance.

Note: The form is written in the masculine form, but it is directed to both sexes equally.

| **Part One: Characteristics of the study participants** | | | | | | | | | | | | | | | | | | | | |
| --- | --- | --- | --- | --- | --- | --- | --- | --- | --- | --- | --- | --- | --- | --- | --- | --- | --- | --- | --- | --- |
| Age: | | Height: | | | Weight: | | | | | | Gender: Male | | | | | | | | Female | |
| Marital status: | | Married | | | Single | | | | | | Divorced | | | | | | | | | |
| Smoking status: | | Smoker | | | Nonsmoker | | | | | | Quit smoking | | | | | | | | | |
| Current residency of student family: | | | | | Village | | | | | | City | | | | | | | Camp | | |
| Place of residence: | | West Bank | | | Jerusalem | | | | | | The occupied area of Palestine | | | | | | | | | |
| Place of current residence during university study: | | Alone in student’s apartment | | | With the family | | | | | | With other students at student’s apartment | | | | | | | | | |
| Academic level: | | Bachelor | | | Master | | | | | | | | | | | | | | | |
| Academic level for bachelor students: | | | | First year | Second year | | | | | | Third year | | | | Fourth year | | | | | |
| Monthly salary income (NIS): | | | Less than 2000 | | 2001-5000 | | | | | | 5001-10000 | | | | | | More than 10000 | | | |
| Chronic diseases: | | HTN | | | DM | | | | | | Others: | | | | | | | | | |
| Current chronic medication: | | Yes | | | No | | | | | | If yes, Specify: | | | | | | | | | |
| **Part Two: Measuring the student’s level of Knowledge about steroid hormones** | | | | | | | | | | | | | | | | | | | | |
| Do these compounds cause acne? | | | | | | | | | Yes | | | No | | | | | | I don’t know | | |
| Do these compounds lead to hair loss? | | | | | | | | | Yes | | | No | | | | | | I don’t know | | |
| Do these compounds lead to infertility? | | | | | | | | | Yes | | | No | | | | | | I don’t know | | |
| Do these compounds increase the risk of heart and arterial diseases? | | | | | | | | | Yes | | | No | | | | | | I don’t know | | |
| Is it FDA approved to use these compounds to increase body mass? | | | | | | | | | Yes | | | No | | | | | | I don’t know | | |
| Does stopping the use of these compounds lead to depression? | | | | | | | | | Yes | | | No | | | | | | I don’t know | | |
| Does stopping the use of these compounds lead to muscle atrophy? | | | | | | | | | Yes | | | No | | | | | | I don’t know | | |
| Does stopping the use of these compounds lead to a decline in physical ability? | | | | | | | | | | | Yes | No | | | | | | I don’t know | | |
| What is the source of your information about these compounds? | | | | | | Friends | | Colleagues | | | | Coach | | | | Social Media | | | | Internet |
|  |  |  |  |  |  | Pharmacist | | Doctor | | | | TV | | | | Others: | | | | |
| **Part Three: Measuring the extent of student’s use of steroid hormones** | | | | | | | | | | | | | | | | | | | | |
| Do you use (or have you ever used) any minerals or vitamins? | | | | | | | | | | Yes | | | | | No | | | | | |
| Have you ever used steroidal hormones for body building? | | | | | | | | | | Yes | | | | | No | | | | | |
| Are you currently using steroidal hormones for body building? | | | | | | | | | | Yes | No | | If yes, Specify: | | | | | | | |
| Do you plan to use steroidal hormones for body building in the future? | | | | | | | | | | Yes | | | | | No | | | | | |
| Do you know what type of product you used? | | | | | | | | | | Yes | | | | | No | | | | | |
| Your source for obtaining these compounds? | | | | | Online | | Pharmacy | | | | Coach | | | | Teacher | | | | Black Market | |
|  |  |  |  |  | Others: | | | | | | | | | | | | | | | |
| Have you consulted any health professional before use? | | | | | | | | | | | | | | Yes | | | | | No | |
| Did you feel an increase in body mass and physical performance after using it? | | | | | | | | | | | | | | Yes | | | | | No | |
| Do you advise (or have you previously advised) one of your colleagues or friends to take it? | | | | | | | | | | | | | | Yes | | | | | No | |
| What are your reasons for using these compounds? | Increase physical activity | | | | | | Treat or prevent an injury related to physical activity | | | | | | | | | | | | | |
|  | Improving the appearance of the body | | | | | | Others: | | | | | | | | | | | | | |
